# Genome sequence of *Umezawaea* sp. strain Da 62-37 isolated from the rhizosphere of *Deschampsia antarctica* E.Desv. (Galindez Island, maritime Antarctic)

**DOI:** 10.1128/mra.00019-24

**Published:** 2024-03-19

**Authors:** Ivan Roman, Stepan Tistechok, Oleksandr Gromyko

**Affiliations:** 1 Genetic and Biotechnology Department, Ivan Franko National University of Lviv, Lviv, Ukraine; 2 The Culture Collection of Microorganisms – producers of antibiotics, Ivan Franko National University of Lviv, Lviv, Ukraine; University of Southern California, Los Angeles, California, USA

**Keywords:** *Umezawaea* sp., Antarctic actinomycetes, genomic DNA

## Abstract

In this study, we present the genome sequence of *Umezawaea* sp. strain Da 62-37, which was isolated from the rhizosphere of *Deschampsia antarctica* E.Desv. (Galindez Island, maritime Antarctic). The *de novo* assembly produced one contig, with a length of 11,793,683 bp. AntiSMASH analysis indicated 49 biosynthetic gene clusters.

## ANNOUNCEMENT

The genus *Umezawaea* is a rare genus of the family Pseudonocardiaceae (1). Members of this genus are promising producers of biologically active compounds, particularly formamicin (2), umezawamides A-B (1), and umezawaones A-D (3). Strain *Umezawaea* sp. Da 62-37 was isolated from the Antarctic region (4). This geographical isolation and high selective environmental pressure can lead to the formation of unique adaptation mechanisms, including biologically active compounds with unusual mechanisms of action.

The *Umezawaea* sp. Da 62-37 strain was isolated from the rhizosphere of *Deschampsia antarctica* (Galindez Island, maritime Antarctic) by direct inoculation of a homogenate of soil samples in sterile water on oatmeal medium and incubated at 28°C for 2–3 weeks (5). For total DNA extraction, *Umezawaea* sp. Da 62-37 strain was inoculated into 20 mL of TSB medium and incubated at 28°C for 72 hours. Genomic DNA was extracted from separate inocula for each sequencing technology using the DNeasy PowerLyzer Microbial Kit (Qiagen, Valencia, CA, USA). For genome sequencing, Illumina genomic shotgun libraries were prepared using a PCR-free protocol using the manufacturer’s instructions (Illumina, Inc.). Sequencing was done on the Illumina Novaseq 6000 system in 2 × 150 bps read mode. Illumina data assembly was done using Newbler v3.0 (6). For long reads sequencing, Oxford Nanopore sequencing technology was used (Oxford, UK). Genomic libraries were prepared from high molecular weight DNA using SQK-LSK110 Kits (Oxford, UK). For reads shearing for Nanopore ligation library preparation, NucleoSpin Genomic DNA Prep Kit (Macherey-Nagel, Germany) was used. Library was sequenced on a single Flongle flow-cell (R10.4.1) with 24 hours run. Base-calling was done using Guppy v5.0.11 with dna_r9.4.1_450bps_sup model (7). ONT data assembly was done with Flye v.2.9 b1774 (8). The reads QC for both Nanopore and Illumina was done using FASTQC (9), and adaptor removal done using AdapterRemoval v2 (10). The number of raw reads generated was 12,790,522 for Illumina and 275,008 for Oxford Nanopore (*N*_50_ 23,596). Initial three rounds of consensus sequence polishing were done using medaka v.1.4.4 (11). After that the polishing with Illumina raw data was done using PILON v.1.24 (12). The sequencing and assembly of the genome were performed by Explogen LLC (Lviv, Ukraine). Default parameters were used for all software unless otherwise specified.

After assembling the genome of *Umezawaea* sp. Da 62-37 contig with a length of 11,793,683 bp was obtained (Table 1). Circulation of the contig was not performed. The GC content was 70.5%, and genome coverage was 81.0×. Completeness and contamination analysis was performed using the CheckM (v1.2.2) (13). The annotation was performed using the NCBI Prokaryotic Genome Annotation Pipeline version 6.6 (14). Genome of *Umezawaea* sp. Da 62-37 contains 10, 321 protein-coding genes, 83 tRNA and rRNA genes, 10 non-coding RNAs, and 157 pseudogenes.

**TABLE 1:** Genomic features of *Umezawaea* sp. Da 62-37 isolated from Antarctic maritime

Parameter	Value
Total length (bp)	11,793,683
No. of contigs	1
GC content (%)	70.5
Size of longest scaffold (bp)	11,793,683
*N*_50_ (bp)	11,793,683
*L*_50_	1
No. of protein-coding genes	10,321
No. of rRNAs	12
No. of tRNAs	61
Estimated completeness (%)	99.5
Estimated contamination (%)	3

The analysis of biosynthetic gene clusters using antiSMASH version 7.1.0 (15) allowed to identify 49 putative clusters. Further research on these clusters could lead to the discovery of new compounds, including antibiotics, and help understand adaptation strategies to extreme environmental factors.

## Data Availability

This study project is available in NCBI under accession number CP135965, BioProject accession number PRJNA1018566, and BioSample accession number SAMN37442707. The raw reads were deposited in the Sequence Read Archive (SRA) as SRR27420182 and SRR27446697.
